# The burrow behavior and influenced factors of a prairie subterranean zokor (*Myospalax psilurus*)

**DOI:** 10.1002/ece3.4705

**Published:** 2018-12-07

**Authors:** He‐Ping Fu, Shuai Yuan, Du‐Hu Man, Xiang‐Xian Chai, Su‐Wen Yang, Dar‐Han Bao, Xiao‐Dong Wu

**Affiliations:** ^1^ Inner Mongolia Agricultural University Hohhot China; ^2^ Key Laboratory of Prataculture and Grassland Resource Ministry of Education Hohhot China; ^3^ Key Laboratory of Forage Cultivation, Processing and High Efficient Utilization of the Ministry of Agriculture Hohhot China

**Keywords:** behavior, meadow steppe, zokor

## Abstract

The Transbaikal zokor (*Myospalax psilurus*) is a dominant rodent distributed in the meadow steppe of Inner Mongolia in northern China. Due to long history of evolution in subterranean environment, the zokor has an adaptive behavior: sealing burrow entrances. When a burrow is damaged, exposed entrances appear, and within a relatively short time, the zokor would be active in sealing the entrances to reduce risks to its survival. In general, it is thought that zokors avoid light and wind, which is consistent with their behavior of sealing burrow entrances. However, direct evidence from field experimental research has been lacking. This study set up 68 field sampling points in a meadow steppe in Inner Mongolia from August to September, 2014 and used a wind–light isolator to study the effects of wind and light factors on zokor burrow entrance sealing behavior. The results showed that there were no significant correlations between wind or light factors and the frequency of zokor burrow entrance sealing. Therefore, wind and light factors are not direct factors associated with zokors actively sealing burrow entrances.

## 1 INTRODUCTION

The zokor is a subterranean rodent that feeds on plant underground organs. There are seven species of zokor in grassland, farmland, and forests in northern China, that is, the Chinese zokor (*Eospalax fontanieri* Milne‐Eedwards 1867),the Rothschild's zokor (*Eospalax rothschildi* Thomas 1911), the Qingling Mountain zokor (*Eospalax rufecens *Allen 1909), the Smith's zokor (*Eospalax smithii *Thomas 1911), the Siberian zokor (*Myospalax myospalax* Laxmann 1773), the Steppe zokor (*Myospalax aspalax *Pallas 1776), and the Transbaikal zokor (*Myospalax psilurus* Milne‐Eedwards 1874) (Ellerman, 1956; Li, 1995; Zheng, Jiang, & Chen, 2012; Jiang et al., 2015). These mammals generally have two seasonal activity peaks annually: the breeding season starting in mid‐May to late May and the food storage period beginning in late August. Each period lasts 20–30 days (Liang, Cai, Liang, & Wang, 1982; Tang & Liu, 2014; Zhang & Liu, 2002; Zhao, 1981). In addition to the seasonal activity peaks, there are also two peaks in summer daytime activities. For example, the Plateau zokor (*Myospalax rufecens baileyi* Thomas 1911) is relatively more active from 4:00 a.m. ~8:00 a.m. and 20:00 p.m. ~24:00 p.m. (Tang & Liu, 2014), and the Gansu zokor (*Eospalax fontanieri cansus* Lyon 1907) from 4:00 a.m. ~6:00 a.m. and 22:00 p.m. ~24:00 p.m. (Meng, 2010). Since 2012, according to our field experimental observations, the Transbaikal zokor's daytime activity peaks is 3:00 a.m. ~7:00 a.m. and 17:00 p.m. ~23:00 p.m., with a short activity period occasionally around 13:00 p.m. In addition, the zokor often engaged in activity or ingested foods to the ground at night (Su et al., 1999; Zhao, 1981; Zheng et al., 2012).

The Hulunbuir meadow steppe is the natural habitat of the Transbaikal zokor in Inner Mongolia (Wang et al., 1997). The zokor often reshapes surface soil to form continuous mounds in the process of feeding plant roots during its year‐round subterranean life (Hu, Li, Chen, & Han, 1994). Especially during its peak activity, zokors dig and burrow to ingest foods underground, and dig out the surface soil to form a large number of mounds, which bury the surface vegetation (Zhang & Liu, 2002). In the process of long‐term evolution and adaptation to the environment, different species of zokors have retained sealing burrow entrances as a common behavior. When a burrow is damaged, exposed entrances appear, and within a relatively short time, the zokor would be active in quickly sealing the burrow entrances. However, it is not precisely known what factors prompt the behavior of zokors to seal the entrances. Previous studies speculated that wind and light entering the burrow were the major factors prompting burrow entrances sealing. Yu and Qian (1958) researched the *Myospalax epsilanus* and speculated that wind might be the external factor prompting zokor burrow entrance sealing. Han (1990) researched the Transbaikal zokor and speculated that light had a greater effect on zokor burrow entrance sealing. Seabloom, Reichman, and Gabet (2000) and Kott, Moritz, Šumbera, Burda, and Němec (2014) studied the burrows of subterranean rodents and speculated that although a complex system of underground burrows greatly hindered the spread of the light, if burrows were opened, there would be weak light in the burrows. Some studies tested the influence of different factors including light on blocking behavior. Werner, Nolte, and Provenza (2005) consider that light was the primary cue entraining plugging behavior in the pocket gophers (*Thomomys bottae*). Kott, Sumbera, and Nemec (2010) researched light perception in two species subterranean rodents in tropical area, suggesting that the photopic vision was conserved and that low acuity residual vision played an important role in predator avoidance and tunnel maintenance. Burda, Sumbera, and Begall (2007) deemed that microclimatic parameters, that is, tunnel geometry structure, temperature, humidness in burrows of subterranean rodents were highly relevant with sealing burrow entrances. However, there must be differences on ecological phylogenetically adaptability of subterranean rodents distributed in different geographical regions (Yuan et al., 2016), the influence factors of the plugging burrow behavior would be different. For the zokor distributed in the north meadow steppe of China, whether effect of the wind or light, or combination effects of wind and light on giving zokors a stimulus to seal the opened entrances? In order to obtain direct evidence from field experiments, we studied how wind and light factors determine the burrow entrance sealing behavior of the Transbaikal zokor in the wild natural environment. Our hypothesis was that wind and light factors were not the determinants of the Transbaikal zokor burrow entrance sealing behavior.

## 2 THE STUDY AREA

**Figure 1:**
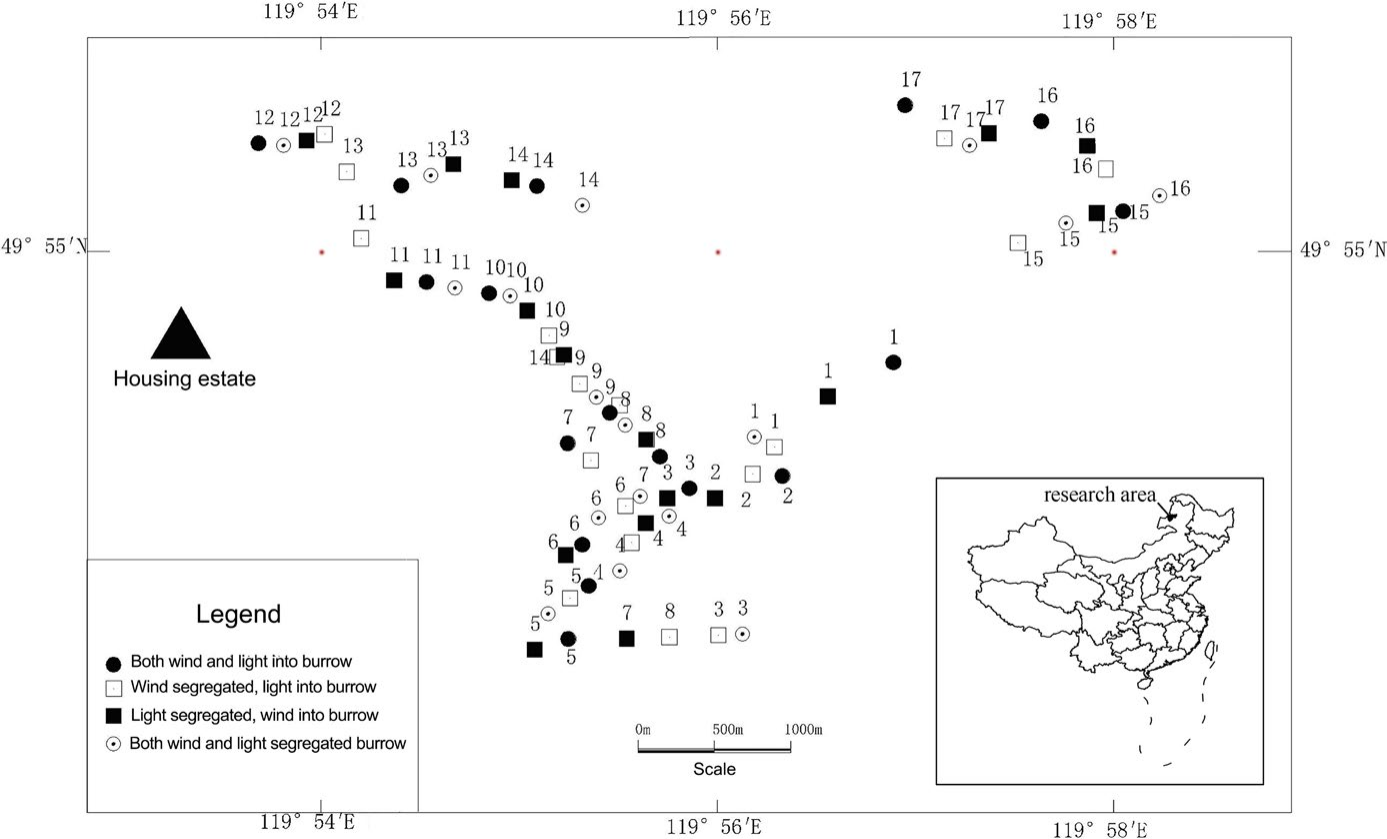
The location of site and sampling plots

The study area was located in the Hulunbuir natural meadow steppe where was located in the northeast of China, and the administrative division belongs to Hulunbuir city of Inner Mongolia Autonomous Region. The geographical position was 119°54′~119°58′E, 49°54′~49°56′N, at an elevation of 696~816 m. The vegetation was mainly the *Stipa baicalensis*, *Sanguisorba officinalis*, *Leymus chinensis,* and *Carex pediformis*, followed by the *Astragalus adsurgens*, *Vicia amoena,* and *Poa ratensis*, and other grasses and herbs. The soil was a chernozem type soil. Data from the nearest weather station for August 2014 show that monthly average temperature was 16.9°C with average wind speed of 4.5 m/s, and maximum wind speed of 9 m/s, and cumulative monthly sunshine of 199.6 hr. The location of site and sampling plots are shown in Figure 1.

## 3 MATERIAL AND METHODS

### 3.1 Apparatus

**Figure 2:**
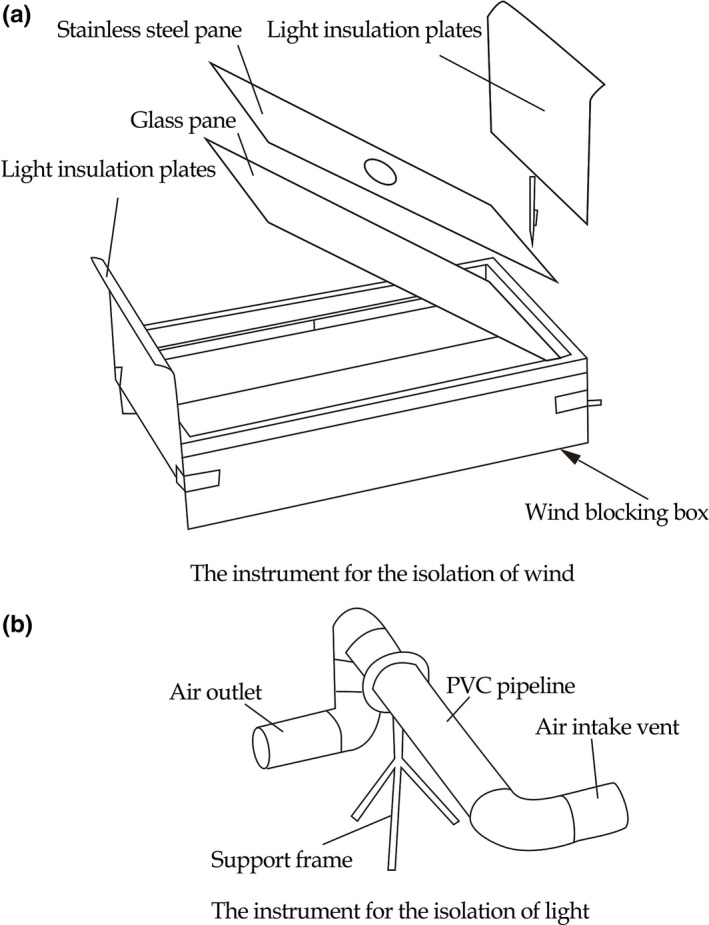
The wind–light isolator for zokor burrows (invention patent number, ZL201410300465.9)

We designed a “wind‐light isolator” (invention patent number, ZL201410300465.9) specifically for the experimental process (Figure 2). The isolator included an apparatus for the isolation of wind (a) and an apparatus for the isolation of light (b). The wind apparatus was made of one piece of glass pane (28 cm × 13 cm × 5 mm), one piece of stainless steel pane (28 cm × 13 cm × 2 mm with a central opening of 110‐mm‐diameter circle through which a PVC pipeline can be inserted), one wind blocking box (30 cm × 16 cm, stainless steel material), and two light insulation plates (20 cm × 15.8 cm × 2 mm, stainless steel material). The light isolation apparatus was composed of a 110‐mm‐diameter PVC pipeline and a support frame. The length of PVC pipeline was 60 cm. The length of air intake vent and air outlet both were 20 cm. When we tested the effect of wind on borrows, the air intake vent and air outlet were oriented horizontal during the experiment, and the air outlet crossed the central hole below the stainless steel pane, ensuring the wind into the tunnel smoothly.

### 3.2 Research design

Research was conducted in August 2014. Before this experiment, we randomly captured and sampled the zokors in this area, and we carried out live tracking for it by wearing a radio tracker (ZAB01, Tianjin Boqian technology co., LTD. China). The zokors can be confirmed that each animal lives in only one system of burrows in the nonbreeding season. Therefore, we observed signs of zokor activity aboveground to select 68 individual zokors as the sample. The gender and age of the zokors were not identified, as the individuals were only observed but not captured. These zokors and their burrow systems were divided into 17 groups. Each group included four treatments: wind without light, light without wind, no wind and no light, and wind with light. The no wind and no light group was the control. Each individual was allocated to only one treatment. We recorded change in wind and light, and the frequency of burrow entrance sealing for each test‐group (four data in each test‐group), and compared them with those of the control group. It was not possible to record data blindly because our study involved focal animals in the field.

### 3.3 Methods

Readers should note that a preliminary or similar version of these results has previously been published in Chinese (Chai et al., 2016). This paper considerably expands on this earlier paper, as well as correcting certain aspects and being published in the English language.

Wind with light: The burrow entrance was bare. After the wind blocking box was inserted into the soil above the intact upper burrow, the soil in the wind blocking box was dug out, then the device was regained. Both wind and light were let into the burrow.

Light with no wind: After the wind blocking box was inserted into the soil above the intact upper burrow, the two light insulation plates were inserted at both ends of the wind blocking box. At the same time, the soil in the wind blocking box was dug out and the box was covered with a glass pane. Finally, the two light insulation plates were pulled up, so that the passage of wind into the burrow entrance was prevented, but the light could shine into the entrance.

Wind with no light: After the wind blocking box was inserted in the soil above the intact upper burrow, the two light insulation plates were inserted at both ends of the wind blocking box. At the same time, the soil in the wind blocking box was dug out and the stainless steel pane was used to cover the wind blocking box and the PVC pipeline was inserted into the central 110‐mm‐diameter opening. Finally, the two light insulation plates were pulled up, so that light was prevented from entering the burrow, but wind could still enter.

No light and no wind: After the wind blocking box was inserted in the soil above the intact upper burrow, the two light insulation plates were inserted at both ends of the wind blocking box. At the same time, the soil in the wind blocking box was dug out and the box was covered with a glass pane, and the glass pane was sealed covered by soil. Finally, the two light insulation plates were pulled up, and the burrow was restored unblocked.

### 3.4 Field experiment

This study only used burrows where the Transbaikal zokor had produced fresh mounds. The appraisal standard for fresh mounds was that the mounds had formed within the last 7 days, the vegetation coverage rate was zero, and the mounds were soft soil (He et al., 2006). The experimental steps were as follows: (a) plot selection. Centered on the new mounds, we used a probe to identify the burrow, and the burrow ascertained was taken as a plot. The straight line distance between each plot was more than 300 m, ensuring that the plots were on the burrows of different individual zokors (Zhou & Dou, 1990), and each burrow was uniquely allocated to one test‐group. (b) We then checked for burrow entrance sealing. After 24 hr, we checked and recorded any change in the conditions of each plot. All of the zokors were measured in accordance with the national regulation (GB14923‐2010 genetic quality control of mammalian laboratory animals), and only its behavior was observed through the test process with no capture so that the zokors were not injured.

### 3.5 Data analysis

An independence test between wind and light factors was conducted using Shannon Mutual Information Analysis (SMIA) (Ince et al., 2010). The relationship between wind or light as one‐way factors and the frequency of zokor burrow entrance sealing was tested by Chi‐square test (*χ* ^2^‐test) to determine independence and Bayes factors test using SAS 9.2 software.

## 4 RESULTS

### 4.1 Mutual information analysis of wind and light factors

Comparing different treatment groups, the number of observed events was highest (i.e., eight times) for the no wind and no light treatment, while the lowest (i.e., five times) was for the wind with light treatment, and the highest frequency of nonsealing of borrows was 12 times (Table 1). The synergistic effects of wind and light factors on the frequency of zokor burrow entrance sealing were analyzed using SMIA. The mutual information values were 7.22 × 10^−5^ (sealing entrance) and 1.02 × 10^−5^ (nonsealing entrance), respectively. This shows that the probability of zokor burrow entrance sealing would be extremely low under the joint effects of wind and light factors, so the relationship between wind and light was independent.

**Table 1:** The effects of wind and light factors on burrow entrance sealing

Treatments	Statistical summary		Total
	Wind	No wind	
Light	5 (12)	7 (10)	12 (22)
No light	6 (11)	8 (9)	14 (20)
Total	11 (23)	15 (19)	26 (42)

Note

The data are frequency of burrow sealing, and the numbers in brackets is the frequency of nonsealing.

### 4.2 One‐way factor effects of wind or light

We tested the relationship between the frequency of sealing or not sealing and wind or light as single factors by the Chi‐square test (*χ* ^2^‐test). The results were as follows: $\chi_{1}^{2}$ = 0.5604 < $\chi_{0.05,1}^{2}$ = 3.84 (*χ* ^2^‐test—sealing), (*p* = 0.857 > 0.05); $\chi_{2}^{2}$ = 0.0623 < $\chi_{0.05,1}^{2}$ = 3.84 (*χ* ^2^‐test—nonsealing), (*p* = 0.924 > 0.05), respectively. Therefore, there was no significant difference. The probabilities of sealing and nonsealing burrow were 0.3823 and 0.6177, respectively (Table 2). Both Likelihood ratios of sealing and nonsealing were approximately equal 1, and the 95% confidence intervals including 1 were breadth (Table 2). Which explained no significant differences. Therefore, the null hypothesis would not be rejected, that wind and light factors were not the determinants of the Transbaikal zokor burrow entrance sealing behavior.

**Table 2:** Bayes' factor test

	Bayes probability	Likelihood ratio	ASE	*SE*	95% confidence interval
Sealing	0.3823	0.9524	0.7966	1.5613	0.86–6.26
Nonsealing	0.6177	0.9818	0.6208	1.2168	0.78–4.17

Note

ASE: advance standard error.

## 5 DISCUSSION

Animal behavior plays a key role in animal adaptation to the environment and is formed through ecological adaptation over long‐term animal evolution and life history. The behavior of zokors in sealing burrow entrances is closely related to their long history of evolution in subterranean environment. Han (1990) observed that the Transbaikal zokor was afraid of wind, light, and disturbance. Once a burrow was accessible to light or wind, they immediately raised the soil, plugging burrow entrances tightly. On the basis of his observations, he suggested that wind and light factors prompted zokors to seal burrow entrances and suggested that light was probably the main factor. Seabloom et al. (2000) dissected the burrows of 19 pocket gophers and found that the angle range of burrow tunnels was from 2° to 30°. The complex structure of burrow tunnels largely limited the spread of light into the burrows. Kott et al. (2014) measured the light spread in artificial and natural burrow tunnels for mole rats (*Fukomys anselli*) and reported that only 0.2%–2.5% of visible light could enter the opened entrances and that the range of light diffusion was limited. Some studies tested the influence of different factors including light on blocking behavior. Coincident conclusion was that light not only induced sealing behavior but also played an important role in burrows maintenance in tropical subterranean rodents. (Kott et al., 2010; Werner et al., 2005). Zhang and Liu (1994) dissected the Gansu zokor and found as the retina thinned, photoreceptor cells decreased dramatically, and that the eyes only had the function of light detection photosensitization but could not form image. The high‐level convergence phenomenon of the visual system appeared in morphological structure and function, such as visual system degradation, smell and hearing system is developed (Zhang & Liu, 2002). Kott et al. (2010) suggested that the photopic vision was conserved and low acuity residual vision played a certain function. Therefore, only light that was inside the burrow tunnels within the visual perception range of zokors could trigger them to seal burrow entrances. However, zokors’ home range is commonly from 10 m^2^ to 1,500 m^2^, and the length of the burrow tunnels is up to 225 m (Zhou & Dou, 1990). In addition, due to complex burrow structure, and the high range of angle changes within burrow tunnels, light diffusion is limited (Kott et al., 2014; Seabloom et al., 2000). Therefore, in a large activity range, it would be difficult for zokor to timely and accurately perceive light in the burrow tunnels. However, the results from previous studies suggested that subterranean rodents probably patrolled their burrow systems regularly, so that they could find damaged parts easily and quickly (Rado, Shanas, Zuri, & Terkel, 1993; Rado, Terkel, & Wollberg, 1998; Skliba et al., 2016; Skliba, Sumbera, Chitaukali, & Burda, 2009; Zuri & Terkel, 1996), and they spent 50% of the time in the daytime in year‐round (Zuri & Terkel, 1996). Accordingly, we would presume that light or wind was not a direct factor prompting sealing burrows to subterranean rodents. In this study, the Transbaikal zokor also sealed burrows under the condition of no light and no wind. This result in an aspect indicated the frequency of the zokor sealing burrow entrances was relevant closing to patrol burrows.

Because oxygen content was low within the enclosed burrow system of subterranean rodents (Band, Malik, Joel, & Avivi, 2012), some researchers have suggested that subterranean rodents have adapted to the hypoxic environment (Avivi et al., 2005; Larson, Drew, Folkow, Milton, & Park, 2014; Tomasco, Rѓo, Iturriaga, & Bozinovic, 2010). Wei, Wei, Zhang, and Yu (2006) found that oxygen pressure in the blood of the Plateau zokor was 1.5 times that of the Plateau pika (*Ochotona curzoniae*). Yu and Qian (1958) inferred that light factors had no effects on zokor entrance sealing behavior and suggested that the wind factor might be the direct factor prompting such behavior. The basic hypothesis of above studies was that the oxygen content increased changing the hypoxic environment of zokors when wind passes into the burrow, or that zokors are sensitive to the high oxygen content in the burrows, thus prompting them to seal entrances. However, Roper, Bennett, Conradt, and Molteno (2001) compared concentrations of CO_2_ and O_2_ in burrows with ambient of both subterranean rodent species. Sumbera, Chitaukali, Elichova, Kubova, and Burda (2004) compared temperatures in burrows with ambient of silvery mole rat. Consistent conclusion was that all measurements of different subterranean rodents indicated mensuration indicators in burrows not differing remarkably to ambient values. In this study, we have observed 35 individuals of the health adult Transbaikal zokor on the ground in the field experiments. Thus, zokors’ activity in the high oxygen environment aboveground was not affected. Our Bayes factors test results showed that the effect of wind on the frequency of the Transbaikal zokor sealing burrow entrances was not significant. Therefore, the wind factor was not a direct factor influencing zokor burrow entrance sealing behavior.

Another result presented the piston‐air resistance hypothesis that an animal moved in a tunnel acting as a piston, increasing gas pressure in front of it. If there was burrow opening, resistance in propagation of that pushed air column was smaller. The animal can by feedback discover that there was an opening in front of it. This could be recognized by tactile sense (Burda et al., 2007; Burda, Bruns, & Müller, 1990). This study was conducted under wild natural conditions; we selected burrows systems from different home ranges of the Transbaikal zokor, and random setting to apply treatments involving different combinations of wind and light factors. Our results show that neither wind nor light independently is direct factors prompting the Transbaikal zokor to seal burrow entrances. So, what factors did prompt them to seal burrow entrances? The piston‐air resistance hypothesis was similar to the “patrol” tunnel. Both viewpoints could explain sealing behavior.

In this study, we found that zokors still sealed burrow entrances nine times (Table 2) under the conditions of no wind and no light. We considered the wind–light isolator itself can influence zokor sealing behavior. Some researchers revealed that disturbance reactions or communication in subterranean rodents were by soil vibrations or voice transmission (Burda et al., 1990; Heth, Frankenberg, Raz, & Nevo, 1987; Hrouzkova, Dvorakova, Jedlicka, & Sumbera, 2013; Mason, Lai, Li, & Nevo, 2010; Rado et al., 1998; Skliba, Sumbera, & Chitaukali, 2008). Sometimes, mole rat could percept at a distance of up to 6 m from ground (Skliba et al., 2008), and exerted anti‐predatory function behavior or accident avoiding. In addition, subterranean rodents patrolled burrows regularly spending a lot of time (Zuri & Terkel, 1996). Based on the above results, when we operated the wind–light isolator on the ground, the operation process probably has caused disturbances to be felt in zokors. After the device was embedded burrow, they patrolled tunnel finding the changes of burrow architecture (Sumbera et al., 2012). These resulted in those plugging burrows. However, the Bayes factors test show that there was no significant difference sealing probability of no wind and no light group comparing with other treatment groups.

## CONFLICT OF INTEREST

The authors declare that they have no conflict of interest.

## AUTHOR CONTRIBUTION

HPF and SY designed the experiment, led the writing of the manuscript. DHM performed the laboratory experiment. XXC, SWY, and DHB performed the statistical analyses and field sampling. XDW provided funding support, laboratory facilities, and intellectual and scientific guidance. All authors contributed critically to the drafts and gave final approval for publication.

## DATA ACCESSIBILITY

Primary data including total data and sample locations have been deposited in the Dryad Repository. https://doi.org/10.5061/dryad.20305t1.
